# Supplementary material for: Is Unplanned Excision of Soft Tissue Sarcomas Associated with Worse Oncological Outcomes?—A Systematic Review and Meta-Analysis
Source: Cancers (Basel). 2024 Jan 19;16(2):443. doi: 10.3390/cancers16020443 (PMC10814439; doi:10.3390/cancers16020443)
Supplement: Supplementary file 1 [file cancers-16-00443-s001.zip › Supplementary Figures.pdf]

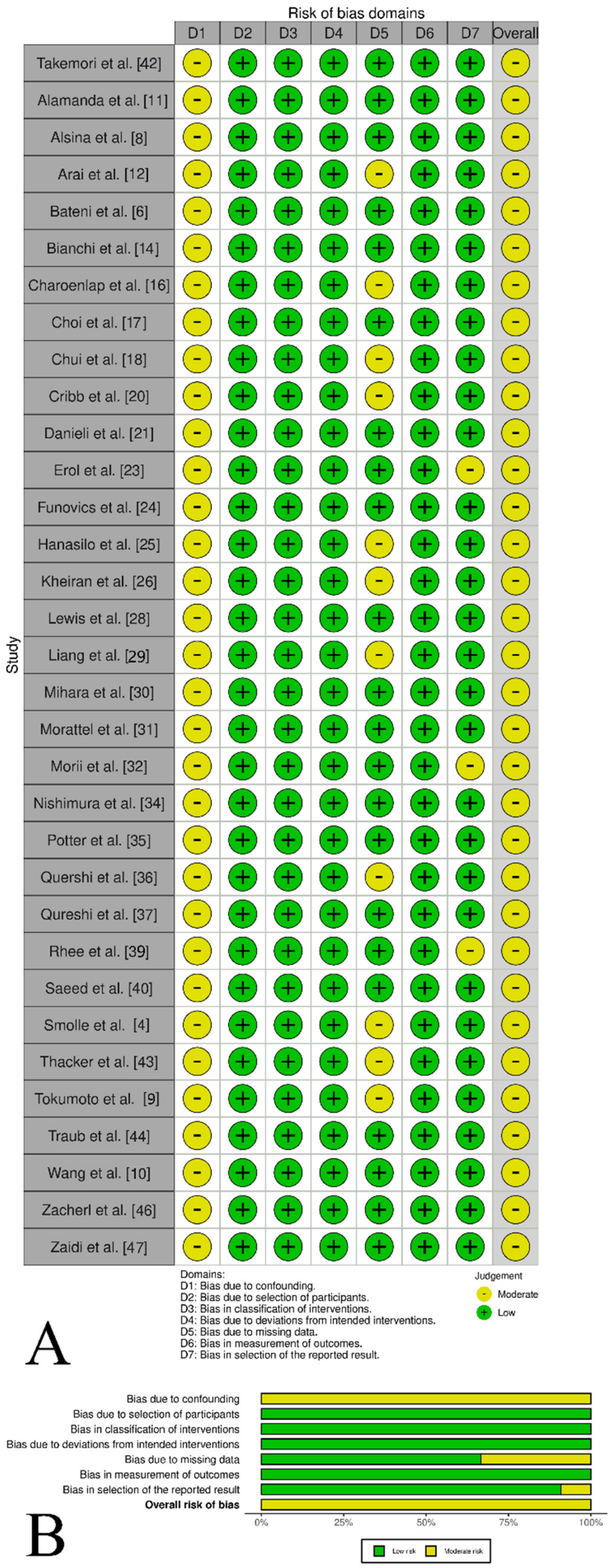

Figure S1. Risk of bias assessment using the Risk of Bias in Non-Randomized studies of Interventions (ROBINS-I) tool represented graphically using the Robvis online visualization tool (A,B).

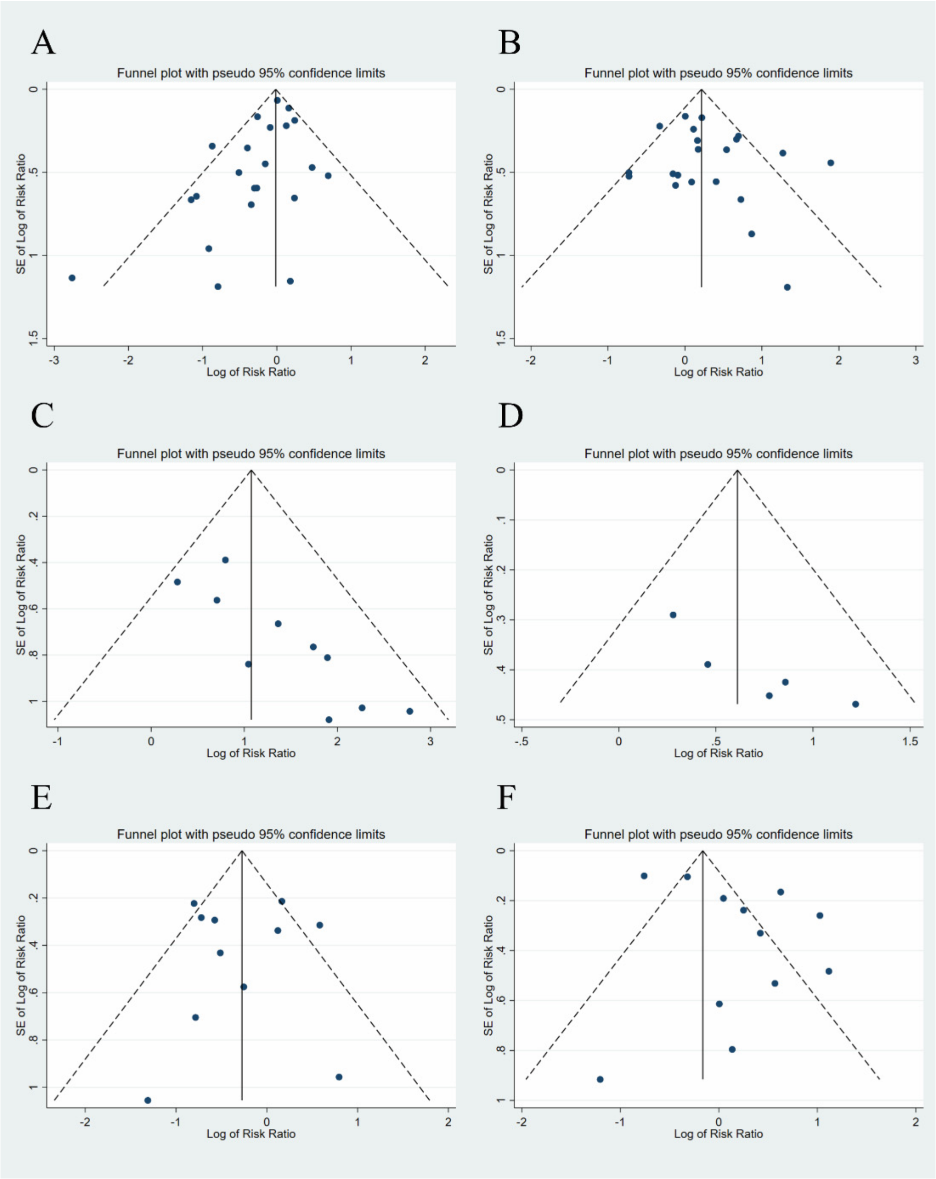

Figure S2. Funnel plots of all studies assessing (A) five-year overall survival, (B) five-year local recurrence-free survival in unplanned excision and planned excision patients, (C) five-year local recurrence in patients with positive and negative residual disease, (D) five-year overall survival in patients that did and did not develop local recurrence, (E) amputation rate and (F) plastic reconstruction surgery rate in unplanned excision and planned excision patient.
