# Supplementary material for: Is Unplanned Excision of Soft Tissue Sarcomas Associated with Worse Oncological Outcomes?—A Systematic Review and Meta-Analysis
Source: Cancers (Basel). 2024 Jan 19;16(2):443. doi: 10.3390/cancers16020443 (PMC10814439; doi:10.3390/cancers16020443)
Supplement: Supplementary file 1 [file cancers-16-00443-s001.zip › Supplementary Table S1.pdf]

Supplementary Table S1. Patients receiving (neo)-adjuvant radiation treatment and/or chemotherapy by group.

Author	Radiation therapy (%)		Chemotherapy (%)	
	PE group [a]	UE group [a]	PE group [a]	UE group [a]
Alamanda et al. [11]	Neoadj.: 14.5<br>Adj.: 44.2<br>Both: 15.9	Neoadj.: 8.5<br>Adj.: 50<br>Both: 5.7	U: 19.2	U: 17.9
Alsina et al. [8]	-	-	-	-
Arai et al. [12]	U: 19	U: 13	U: 38	U: 18
Arai et al. [13]	-	U: 8	-	U: 15
Bateni et al. [14]	Adj.: 48	Adj.: 42.8	Adj.: 6.8	Adj.: 6.1
Bianchi et al. [15]	U: 76	Adj: 100	Neoadj.: 15.2<br>Adj.: 21.8	Neoadj.: 2<br>Adj.: 16
Charoenlap et al. [16]	Adj.: 89	Adj.: 84.5	Adj.: 4.1	Adj.: 6.2
Choi et al. [17]	Adj.: 60	Adj.: 63	Adj.: 62	Adj.: 47
Chui et al. [18]	-	-	-	-
Collin et al. [19]	-	-	-	-
Cribb et al. [20]	Adj.: 36.4	Adj.: 62.5	Adj.: 9.1	Adj.: 0
Danieli et al. [21]	U: 56.5	U: 33.9	U: 35.9	U: 17
Davis et al. [3]	-	-	-	-
Emrich et al. [22]	-	-	-	-
Erol et al. [23]	Neoadj.: 6.7<br>Adj.: 77.3	Neoadj.: 77.3<br>Adj.: 78	Neoadj.: 8<br>Adj.: 49.3	Neoadj.: 6<br>Adj.: 44
Funovics et al. [24]	Adj.: 74	Adj.: 66.8	Adj.: 62.7	Adj.: 46.7
Hanasilo et al. [25]	Adj.: 62	Adj.: 47.8	Adj.: 72.4	Adj.: 52.1
Kheiran et al. [26]	-	-	-	-
Koulaxouzidis et al. [27]	-	-	-	-
Lewis et al. [28]	-	-	-	-
Liang et al. [29]	-	-	-	-
Mihara et al. [30]	-	-	-	-
Morattel et al. [31]	-	-	-	-
Morii et al. [32]	-	-	U: 29	U: 25
Nakamura et al. [33]	-	-	-	-
Nishimura et al. [34]	U: 22.2	U: 40	U: 33.3	U: 40
Potter et al. [35]	Adj.: 58	Adj.: 64	Adj.: 60	Adj.: 38
Quershi et al. [36]	U: 45.8	U: 62.8	U: 29	U: 31.4
Qureshi et al. [37]	-	-	-	-
Rehders et al. [38]	-	-	-	-
Rhee et al. [39]	Neoadj.: 100	Neoadj.: 100	-	-
Saeed et al. [40]	Neoadj.: 77.2<br>Adj.: 11.4	Neoadj.: 32.3<br>Adj.: 17.7	U: 32	U: 3
Scoccianti et al. [41]	-	-	-	-
Smolle et al. [4]	Adj.: 51.7	Adj.: 59.4	Adj.: 33.3	Adj.: 24.2
Takemori et al. [42]	Adj.: 9.1	Adj.: 8.33	Adj.: 10	Adj.: 20.8
Thacker et al. [43]	Adj.: 8.7	Adj.: 55	-	-
Tokumoto et al. [9]	Adj.: 27	Adj.: 16.2	Adj.: 28.2	Adj.: 18.1
Traub et al. [44]	Adj.: 85.7	Adj.: 85.1	Adj.: 8.6	Adj.: 6.4
Ueda et al. [45]	-	-	-	-
Wang et al. [10]	U: 41.4	U: 50	U: 14.3	U: 20.5
Zacherl et al. [46]	Adj.: 57.1	Adj.: 71.4	Neoadj.: 21.4<br>Adj.: 37.5	Neoadj.: 10.7<br>Adj.: 25
Zaidi et al. [47]	Neoadj.: 27<br>Adj.: 23	Neoadj.: 9<br>Adj.: 28	Neoadj.: 18	Neoadj.: 6
Zhao et al. [48]	-	-	-	-

[a] The values displayed in these columns refer to the proportion of patients receiving such treatment (out of the total number of patients in the group).
Adj.: adjuvant; *Neoadj.*: neoadjuvant; *PE*: planned excision; *U*: unspecified; *UE*: unplanned excision.
