# Supplementary material for: Is Unplanned Excision of Soft Tissue Sarcomas Associated with Worse Oncological Outcomes?—A Systematic Review and Meta-Analysis
Source: Cancers (Basel). 2024 Jan 19;16(2):443. doi: 10.3390/cancers16020443 (PMC10814439; doi:10.3390/cancers16020443)
Supplement: Supplementary file 1 [file cancers-16-00443-s001.zip › Supplementary Table S2.pdf]

Supplementary Table S2. Location and histology of soft tissue sarcomas by group among included studies.

Author	UEx (%) [a]	LEx (%) [a]	Trunk (%) [a]	Most common soft tissue sarcoma histology by group (%)	
				PE group	UE group
Alamanda et al. [11]	20.9 / 39.6	79.1 / 60.4	0 / 0	UPS (36.6), LPS (32.6), LMS (8.1)	UPS (35.9), SS (14.2), FS (11.3)
Alsina et al. [8]	18.8 / 24.1	74.2 / 60	2.9 / 11.8	UPS (32.5), LPS (27), SS (10.4)	UPS (31.7), LPS (14.5), SS (12.4)
Arai et al. [12]	-	-	-	LPS (29.7) UPS (17.2), MPNST (10.2)	UPS (25.4) LPS (15.9), DFSP (14.3)
Arai et al. [13]	- / 18.6	- / 48.7	- / 32.7	-	UPS (34.5), LPS (19.5), DFP (10.6)
Bateni et al. [14]	19.6 / 24	41.2 / 36.2	46.3 / 38.8	UPS (30.6), LPS (24.3), Sarcoma NOS (20.9)	UPS (31.6), LPS (19.9), Sarcoma NOS (17.8)
Bianchi et al. [15]	25 / 23	75 / 77	0 / 0	UPS (28), Fibroblastic/MFS (24), LPS (16)	UPS (34), SS (17), Fibroblastic/MFS (17)
Charoenlap et al. [16]	-	-	10 / 13.7	UPS (36.9), LPS (16.5), SS (7.9)	UPS (37.9), LMS (12.4) SS (11.2)
Choi et al. [17]	12 / 29	88 / 71	-	-	-
Chui et al. [18]	-	-	5.3 / 11.7	SS (23.3), UPS (23.3), FS (16.7)	SS (36.8), UPS (10.5)
Collin et al. [19]	-	-	-	-	-
Cribb et al. [20]	-	100 / 100	-	SS (27.3), CCS (18.2)	SS (56.3), MFS (12.5)
Danieli et al. [21]	23.5 / 28.9	69.1 / 56.9	7.4 / 14.2	UPS (19.1), MFS (17.4), LPS (12.0)	MFS (15.7), LPS (13.3), UPS (13)
Davis et al. [3]	-	-	-	-	-
Emrich et al. [22]	-	-	-	-	-
Erol et al. [23]	16 / 32	82.7 / 66	1.3 / 2	-	-
Funovics et al. [24]	16.6 / 26.3	64.9 / 56.4	18.5 / 17.4	LPS (14.6), SS (13.5), LMS (12.4)	LPS (15.8), SS (13.5), UPS (13.5)
Hanasilo et al. [25]	-	-	-	-	-
Kheiran et al. [26]	-	100 / 100	-	SS (42.1), LMS (15.8)	LMS (42.9), SS (28.6)
Koulaxouzidis et al. [27]	- / 31	- / 47.9	- / 19.7	-	LPS (37.3), UPS (21.7), FS (13.3)
Lewis et al. [28]	-	-	-	LPS (27.9), UPS (24.4), SS (12.3)	UPS (33.9), LPS (22.4), SS (13)
Liang et al. [29]	-	-	39.8 / 36.4	-	-
Mihara et al. [30]	17 / 19	59 / 53	27.3 / 90.6	-	-
Morattel et al. [31]	10.9 / 23.4	87.6 / 70.3	1.2 / 6.3	Sarcoma NOS (29.9) LPS/ALT (29.1), SS (8.6)	Sarcoma NOS (25), MFS (18.7) LPS/ALT (17.2),
Morii et al. [32]	16.2 / 25	67.6 / 58.3	16.2 / 16.7	-	-
Nakamura et al. [33]	-	-	-	-	UPS (25.9), LPS (18.8), MFS (13.2)
Nishimura et al. [34]	-	-	-	-	SS (60), CCS (20), RMS (20)
Potter et al. [35]	-	-	-	-	-
Quershi et al. [36]	-	-	-	-	-
Qureshi et al. [37]	17.2 / 29.9	66.5 / 46.3	15.8 / 18.6	LPS (25.8), UPS (18.2), MFS (14.4)	LMS (17.9), LPS (14.9), MFS (11.2)
Rehders et al. [38]	- / 22	- / 51	- / 27	-	LPS (28), UPS (26), LMS (11)
Rhee et al. [39]	11.8 / 43.5	76.5 / 47.8	11.8 / 8.7	MFS (100)	MFS (100)
Saeed et al. [40]	23.7 / 38.2	76.3 / 61.8	-	UPS (24.1), LPS (32.2), SS (18)	LPS (26.5), SS (20.6), UPS (17.6),
Scoccianti et al. [41]	- / 32.8	- / 55.7	- / 8.4	-	LMS (17.6), UPS (14.5), LPS (14.5)
Smolle et al. [4]	16.33 / 24.9	69.4 / 52.3	11.3 / 17.1	LPS (28.1), MFS (22.4), LMS (9.8)	LPS (20.3), MFS (21.7), LMS (11.4)
Takemori et al. [42]	17.3 / 12.5	60 / 58.3	12.7 / 20.8	UPS (18.2), MFS (13.6), LMS (12.7)	MFS (25), UPS (20.8), LMS (16.7)
Thacker et al. [43]	-	-	-	-	-
Tokumoto et al. [9]	-	-	-	-	UPS (30), MFS (15), DFSP (11)
Traub et al. [44]	20.5 / 24.4	79.6 / 75.5	0 / 0	UPS (41.4), LPS (16.5), MFS (7.1)	UPS (46.8), LPS (13.8), MPNST (10.6)

Ueda et al. [45]	-	-	-	-	-
Wang et al. [10]	-	-	-	-	-
Zacherl et al. [46]	-	-	-	-	-
Zaidi et al. [47]	-	-	15 / 22	-	-
Zhao et al. [48]	-	-	29.3	-	-

[a] The values displayed in these columns refer to patients with planned excision (first number) and with unplanned excision (second number).

ALT: atypical lipomatous tumor; *CCS*: clear cell sarcoma; *DFSP*: dermatofibrosarcoma protuberans; *FS*: fibrosarcoma; *LEx*: lower extremity; *LMS*: leiomyosarcoma; *LPS*: liposarcoma; *MFS*: myxofibrosarcoma; *MPNST*: malignant peripheral nerve sheath tumor; *NOS*: not otherwise specified; *PE:* planned excision; *RMS*: rhabdomyosarcoma; *SS*: synovial sarcoma; *UE:* unplanned excision; *UEx*: upper extremity; *UPS*: undifferentiated pleomorphic sarcoma.
