# Supplementary material for: Is Unplanned Excision of Soft Tissue Sarcomas Associated with Worse Oncological Outcomes?—A Systematic Review and Meta-Analysis
Source: Cancers (Basel). 2024 Jan 19;16(2):443. doi: 10.3390/cancers16020443 (PMC10814439; doi:10.3390/cancers16020443)
Supplement: Supplementary file 1 [file cancers-16-00443-s001.zip › Supplementary Table S3.pdf]

Supplementary Table S3. Quality Assessment Using Strengthening the Reporting of Observational Studies in Epidemiology (STROBE) Checklist Items.

First Author (Year)	Methods					Results				Discussion	Score	Included
	5 - Setting	6 - Participants	7 - Variables	8 - Data sources	12 - Statistical Methods	13 - Participants	14 - Descriptive data	15 - Outcome data	16 - Main results	19 - Limitations		
Arai et al [13]	2	2	2	1	2	1	1	2	2	2	17	Yes
Collin et al [19]	2	2	2	1	1	2	2	2	2	2	18	Yes
Davis et al [3]	2	2	2	1	2	1	1	2	2	1	16	Yes
Emrich et al [22]	2	2	1	2	2	1	1	2	2	1	16	Yes
Koulaxouzidis et al [27]	2	2	2	2	1	1	1	2	2	2	17	Yes
Nakamura et al [33]	2	2	1	1	2	2	2	2	2	2	18	Yes
Rehders et al [38]	2	2	2	1	2	1	2	1	1	2	16	Yes
Scoccianti et al [41]	2	2	2	2	1	1	1	2	2	1	16	Yes
Ueda et al [45]	2	2	2	2	2	1	2	1	1	2	17	Yes
Zhao et al [48]	2	2	1	2	1	2	2	2	2	2	18	Yes
